# Comparison of inflammatory markers in induced and spontaneous sputum in a cohort of COPD patients

**DOI:** 10.1186/s12931-014-0138-6

**Published:** 2014-11-15

**Authors:** Solveig Tangedal, Marianne Aanerud, Louise JP Persson, Karl A Brokstad, Per S Bakke, Tomas M Eagan

**Affiliations:** Department of Thoracic Medicine, Haukeland University Hospital, Bergen, Norway; Institute of Clinical Science, Faculty of Medicine, University of Bergen, Bergen, Norway

**Keywords:** COPD, Sputum sampling, Inflammatory markers

## Abstract

**Background:**

Sputum induction is a non-invasive method for obtaining measurements of inflammation in the airways. Whether spontaneously sampled sputum can be a valid surrogate is unknown. The aim of this study was to compare levels of six inflammatory markers in sputum pairs consisting of induced and spontaneous sputum sampled on the same consultation either in a stable state or during exacerbations of chronic obstructive pulmonary disease (COPD).

**Methods:**

433 COPD patients aged 40–76, Global initiative for chronic Obstructive Lung Disease (GOLD) stage II-IV were enrolled in 2006/07 and followed every six months for three years. 356 patients were followed for potential exacerbations. Interleukin-6, interleukin-8, interleukin-18, interferon gamma-inducible protein-10, monokine induced by gamma interferon and tumor necrosis factor-alpha (IL-6, IL-8, IL-18, IP-10, MIG and TNF-α) were measured by bead based multiplex immunoassay in 60 paired sputum samples from 45 patients. Albumin was measured by enzyme immunoassay, for concentration correction. Culturing for bacterial growth was performed on 24 samples. Bland-Altman plots were used to assess agreement. The paired non-parametric Wilcoxon signed-rank test, the non-parametric Spearman’s rank correlation test and Kruskal-Wallis test were used for statistical analyses. For all analyses, a p-value < 0.05 was considered significant.

**Results:**

Agreement between the two measurements was generally low for all six markers. TNF-α was significantly higher in spontaneous sputum at exacerbations (p = 0.002) and trending higher at the steady state (p = 0.06). Correlation coefficients between the levels of markers in induced and spontaneous sputum varied between 0.58 (IL-18) to 0.83 (IP-10). In spontaneous sputum IL-18 and MIG were higher in ex-smokers (p < 0.05). The levels of all markers were higher in GOLD stage III & IV except for IL-6 in spontaneous sputum and IL-18 in induced sputum, compared with GOLD stage II, although not statistically significant. In spontaneous sputum the levels of IL-6 were significantly higher if Haemophilus influenzae (HI) was not cultured.

**Conclusion:**

We observed a low agreement and significant differences in inflammatory markers between induced and spontaneous sputum, both at steady state and exacerbations. We recommend considering sampling method when reporting on inflammatory markers in sputum.

## Background

Chronic obstructive pulmonary disease (COPD) is a chronic inflammatory disease affecting both the airways and lung parenchyma [1]. The increased airway inflammation has been well described, but its role is yet controversial [2]. Obtaining reliable measurements of airway inflammation non-invasively can enable large cohort studies. Biomarkers sampled by methods like exhaled breath condensate and induced sputum have been compared recently [3]. Induced sputum sampling (ISS) is a non-invasive procedure, which has been standardized and used extensively the last 20 years [4]. Nebulized and inhaled saline increases sputum production in the lungs [4]. Induction has been reported to provide sputum samples of sufficient quality for analyses in more than 80% of asthma and COPD patients [5–8]. In patients with obstructive lung disease, ISS is usually performed in the steady state as it can induce bronchoconstriction [9,10]. However, at least one study has shown that it can be done safely also during exacerbations in patients with mild to moderate COPD [11].

An alternative to ISS is spontaneous sputum sampling (SSS). Levels of inflammatory markers and cell counts in spontaneous and induced sputum have been presented without discriminating between the two sampling methods in some studies [12–14]. Two studies have found that cell viability was higher in induced than spontaneous sputum in patients with asthma or COPD [15,16]. However, few studies have addressed whether induced and spontaneous sputum sampled from patients with COPD can actually be used interchangeably for analyses of inflammatory markers, as it was pointed to in a review article published as late as in 2013 [17]. More studies on the subject were recommended already in 2002 [4].

The aim of this study was to compare the levels of the six common inflammatory markers interleukin 6, 8 & 18 (IL-6, IL-8 IL-18), interferon gamma-inducible protein-10 (IP-10), tumor necrosis factor-alpha (TNF-α) and monokine induced by gamma interferon (MIG) in paired induced and spontaneous sputum samples collected from COPD patients in the stable state and/or during acute exacerbations. These markers were chosen for different roles in airways inflammation in COPD, as part of the analyses in the Bergen COPD Exacerbation Study. In addition, this study allowed for an assessment of the safety of sputum induction in COPD patients undergoing an exacerbation.

## Methods and material

### Study population

The Bergen COPD Cohort Study (BCCS) was a three year follow-up of 433 COPD patients from western Norway between 2006 and 2010, previously described in detail [18]. The patients were invited to our study centre every six months, and sputum induction was performed at nearly all visits. Of the 433 COPD patients, 356 patients living in a proximity that meant they belonged to the Bergen hospital district were offered concomitant participation in the Bergen COPD Exacerbation Study (BCES). Patients included in the BCES were given a laminated green-card with detailed instructions regarding potential symptoms of COPD exacerbations and a telephone number to our study nurse. The telephone was open 12 hours per day, seven days a week for the three years the study lasted. Once contact had been made, the study nurse determined whether immediate hospitalization was necessary, or whether a visit with a study physician could be scheduled the next working day. During that visit or at the ward the day after hospitalization, sputum induction was attempted if our study physician determined the event to be a clinical COPD exacerbation, with a formal assessment according to Wedzicha and Donaldsons’s definition [19].

Spontaneous sputum samples were collected before the induced sputum sample at the same time point at occasions when the patients presented with abundant sputum. In total 60 sputum pairs of acceptable quality from 45 patients in the stable state (n = 31) or during COPD exacerbation (n = 29) were available for analysis. Classification into Global initiative for chronic Obstructive Lung Disease (GOLD 2007) stage and information on smoking habits, were based on the baseline visit in the BCCS. All patients provided written informed consent, and both studies were approved by the Norwegian Regional Ethical Committee.

### Sputum sampling and processing

Inductions were performed using an ultrasonic wave nebulizer. Hypertonic saline (3%) was inhaled seven minutes times three, and sputum was attempted sampled after each inhalation. If however, the patient was evaluated by the study physician as being too clinically obstructive, or if the patient did not want to inhale an increased saline concentration, the physiological saline concentration of 0.9% was inhaled instead. Of the 60 sputum pairs evaluated, induction was done with 3% saline in 47 cases, 0.9% in ten cases, while for three inductions the concentration was not recorded. Spirometric evaluations (Vitalograph S-model Vitalograph Ltd., Buckingham, England at regular visits in the steady state, EasyOne model 2001 Ndd Medizintechnik AG, Zurich, Switzerland at exacerbation visits) were performed after inhalation of 200–400 ug salbutamol prior to induction with saline. Spirometry was then repeated after each inhalation of the saline. The procedure ended if FEV_1_ declined 20% or more, if the patient’s symptoms worsened, or if the patient did not wish to proceed. If the patient’s post-bronchodilator oxygen saturation was <90%, induction was not performed.

For the SSS, patients were asked to expectorate in two different cups, and the most purulent sputum was processed. Both types of sputum samples were kept on ice until processed for quality control and storage, usually within 30 minutes. To break disulphide bonds in mucin, 4 ml dithiothreitol 0.1% (DDT) per gram sputum were added [20]. The samples were then homogenized using an Eppendorf homogenizer at 600 rpm for 15 minutes at a temperature of 4 degrees Celsius. Phosphate-buffered saline (PBS) was added, and the sample filtered to increase homogenization. Supernatants were removed after 15 minutes centrifugation at 4 degrees Celsius, 450 *g*, aliqouted in 0.5 ml tubes, and stored at −80 degrees Celsius. Trained personnel evaluated viability after staining with tryptan blue. For the sputum samples to be considered of acceptable quality there had to be > 1 million/mL cells, < 20% epithelial cells and the leucocyte viability had to be > 30%. After December 2006, all sputum samples were also cultured at the Department of Microbiology, Haukeland University Hospital.

The sputum samples were analysed for cytokines using the Luminex® xMAP® technology (Luminex Corporation, Austin, Texas). The cytokine assay used was made by combining standards from BioRad (Bio-Plex Pro Human Cytokine Standards Group I 27-Plex #171-D50001, Lot No 5022130. Bio-Plex Pro Human Cytokine Standards Group II 23-Plex #171-D10502 Lot No 5015357) and singleplex assays containing beads for analyses of IL-6, IL-8 IL-18, IP-10, TNF-α and MIG. Thus, all six markers were analyzed in simplex. The samples were processed on a Luminex 100 instrument and the results collected and stored by STarStation software version 2.0 (STarStation Software Version 2.0, Applied Cytometry, Sheffield, UK.) The procedure was performed according to the manufacturer’s instructions on six separate days in September 2011.

For 58 of the 60 sputum pairs we also had enough material to perform an enzyme immunoassay of levels of albumin in duplex (Albumin Human ELISA kit, ab 108788, Abcam, Cambridge, UK). Albumin was used as a correction factor for concentration differences between the induced and spontaneous sample for each pair in the following way: The induced to spontaneous albumin ratio was calculated for each sputum pair, and the level of each of the six markers in each of the spontaneous sputum samples multiplied by the corresponding ratio. All later statistical analyses were performed both on “corrected” sputum levels and “uncorrected” sputum levels.

### Statistical analyses

Stata 12.0 was used for the statistical analyses (StataCorp. College Station, Texas). Bland-Altman plots were made to assess agreement between the measured levels of the markers in induced and spontaneous sputum pairs. Bland & Altman advocates using the difference between the two measurements as the central measurement of bias, and the spread of the difference as a measure of limits of agreement [21]. Usually the difference between the measurements is plotted against the mean of the two measurements, with 2 standard deviations (SD) of the difference representing the 95% limits of agreement. However, sometimes the difference is dependent upon the size of the mean, in which Bland & Altman advocates plotting on a log scale [22]. This was the case for all six markers in our study.

The inflammatory markers were not normally distributed, hence the paired non-parametric Wilcoxon signed-rank test was used to compare levels of the markers and cell viability between spontaneous and induced sputum. For correlation analyses between spontaneous and induced samples the non-parametric Spearman’s rank correlation test was used. For comparisons of the levels of inflammatory markers by clinical characteristics, Kruskal-Wallis test was used. For comparisons of FEV_1_ decline between stable state and exacerbations during inductions, Wilcoxon signed-rank test was used. For all analyses, a p-value of less than 0.05 was considered significant.

## Results

The characteristics of the study population are presented in Table 1. 60 sputum pairs were available from 45 patients, of which 15 of the patients were women. Of the 60 sputum pairs, 31 were sampled during the stable state and 29 during COPD exacerbations (Table 1).

**Table 1:** **Characteristics of the study population**

	**n**	**%**
***Patients***	45	
*Age, mean (range)* ^*^	63.4 (46–74)	
*Sex*		
Women	15	33
Men	30	67
*Smoking habits*		
Ex	30	67
Current	15	33
*GOLD (2007) stage* ^*^		
II	15	33
III	24	53
IV	6	13
*Patients with one sputum pair* ^**^	36	80
*Patients with multiple sputum pairs*	9	20
***Sputum pairs***	60	
stable state	31	52
during exacerbation	29	48
*H.influenza positive* ^†^		
No	12	
Yes	12	

^*^At inclusion.

^**^Consisting of one spontaneous and one induced sputum sample.

^†^Detected in induced and/or spontaneous sputum sampled from stable state visits, and/or exacerbations.

Mean cell viability was 98% for both the induced and spontaneous sputum samples. Among the induced samples, 2 out of 60 samples had viability below 90%, for the spontaneous samples all were 90% viable or better.

Of the six inflammatory markers, TNF-α was significantly higher when measured in spontaneous sputum during exacerbations and almost reaching statistical significance in the steady state (Table 2). For the other markers, no clear trend was seen (Table 2).

**Table 2:** **A comparison of inflammatory markers in induced and spontaneous sputum sampled from the COPD patients at the time; either during a COPD exacerbation or during the stable state**

	**During a COPD exacerbation n = 28**			**During the stable state n = 30**		
	**Induced sputum sample**	**Spontaneous sputum sample**	p*	**Induced sputum sample**	**Spontaneous sputum sample**	p*
IL-6(pg/ml) median, IQR	10.0(4.9-26.6)	10.4(2.8-23.3)	0.29	20.5(6.11-43.2)	13.9(2.2-55.2)	0.46
IL-8(pg/ml) median, IQR	339.7(193.6-663.9)	344.9(173.9-812.2)	0.77	514.2(225.6-1257.2)	3709.9(171.7-976.7)	0.18
IP-10(pg/ml)	735.0(205.4-2099.6)	372.1(218.7-1416.2)	0.15	529.0(215.4-2554.8)	362.3(142.0-1393.1)	0.07
TNF-α (pg/ml) median, IQR	3.0(0.2-11.8)	6.3(2.0-34.0)	0.002	0.9(0-2.4)	1.3(0.2-4.3)	0.06
IL-18 (pg/ml) median, IQR	9.2(4.9-12.9)	10.6(3.6-27.3)	0.52	6.5(2.4-25.6)	9.6(0.8-18.2)	0.64
MIG (pg/ml) median, IQR	539.2(100.1-1496.0)	384.6(221.2-1997.0)	0.41	534.4(56.9-1450.1)	567.4(178.0-2031.7)	0.82

^*^Wilcoxon sign rank test.

**Figure 1:**
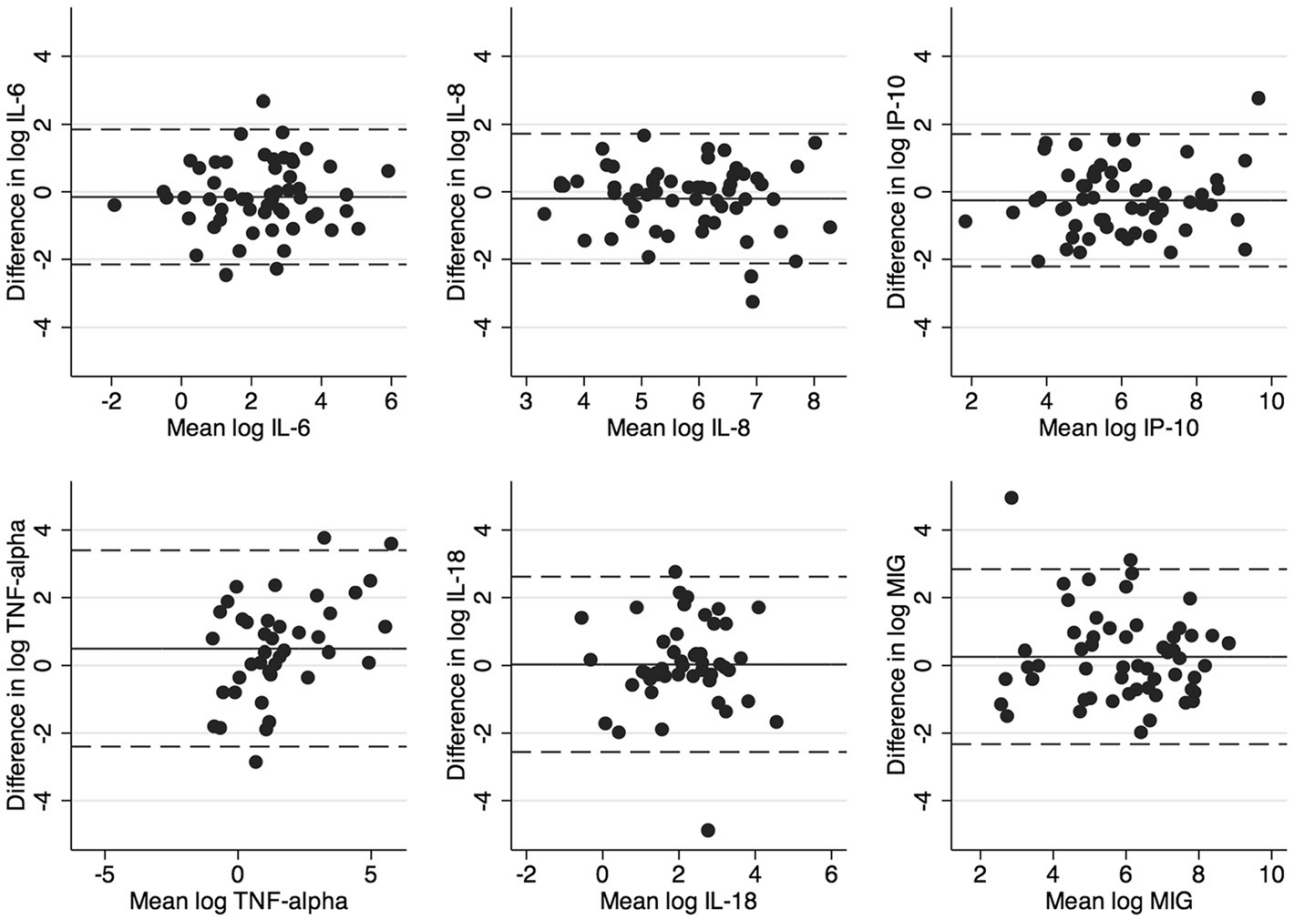
**Bland-Altman plots of the agreement between measurements of six inflammatory markers in induced and spontaneous sputum samples.**

Bland-Altman plots for all six inflammatory markers on the log scale are presented in Figure 1. To obtain the limits of agreement the antilog of the two standard deviations were calculated, and these are presented in Table 3 together with the Spearman’s rank correlation coefficients. Although the correlation was fair, varying between 0.58 for IL-18 to 0.83 for IP-10, the agreement was quite low for all six inflammatory markers. Since the 95% limits of agreement were calculated on the log scale, the upper and lower limits represents ratios relative to one. Thus, based on the calculations presented in Table 3, one would expect the measurement of for instance IL-6 in spontaneous sputum to fall between 6 times higher or 8 times lower than that measured in induced sputum 95% of the time.

**Table 3:** **Rank correlation coefficients and the 95% limits of agreement between measurements of six inflammatory markers in induced and spontaneous sputum samples**

	**Correlation coefficient** ^*^	**Bland & Altmans 95% limit of agreement** ^**†**^	
		**Lower**	**Upper**
IL-6 (pg/ml)	0.729	0.12	6.35
IL-8 (pg/ml)	0.695	0.12	5.59
IP-10 (pg/ml)	0.833	0.08	13.7
TNFα (pg/ml)	0.600	0.11	5.53
IL-18 (pg/ml)	0.583	0.09	29.99
MIG (pg/ml)	0.754	0.10	17.24

^*^Spearman’s rank correlation test.

^†^±2 SD of the mean difference between the two measurements.

Even though agreement between individual measurements was low, there could be value to the spontaneous samples if the levels of the markers showed the same associations to clinical parameters in spontaneous as in the induced sputum samples. Potential associations between measured levels of the inflammatory markers in spontaneous and induced samples, and clinical variables are presented in Table 4. There was no consistent difference in levels of any of the six markers between current and ex-smokers. However, in the spontaneous samples the measured levels of IL-18 and MIG were significantly higher in ex-smokers, an association not found in the induced samples. For all markers except IL-6 and IL-18, there was a non-significant trend of higher levels in GOLD stage III & IV compared with GOLD stage II. Importantly however, the pattern was the same for both spontaneous and induced sputum samples. Finally, in the 24 sputum pairs where culture was obtained, we examined which impact Haemophilus influenzae (HI) had on the pattern of the sputum markers. In the spontaneous samples HI was associated with significantly lower levels of IL-6, a difference not found in the induced samples. In addition, we observed that in the spontaneous samples levels of MIG were lower in sputum with HI, whereas the opposite pattern was seen in the induced samples (Table 4).

**Table 4:** **Median (IQR) values of inflammatory markers in induced and spontaneous sputum samples by smoking, GOLD stage and H. influenzae carrier state**

	**Smoking status**			**GOLD stage**			**H. influenzae**		
	**Current smoker n = 15**	**Ex-smoker n = 29**	p*	**II n = 15**	**III+IV n = 29**	p*	**No n-12**	**Yes = n = 12**	p*
*IL-6(pg/ml)*									
induced	17.2(5.7-36.2)	16.7(4.0-43.2)	0.63	16.7(4.5-67.8)	19.4(5.2-36.2)	0.78	20.7(10.9-49.0)	17.8(1.3-28.6)	0.16
spontaneous	10.2(3.0-29.5)	21.5(5.8-48.2)	0.22	15.6(2.2-64.9)	13.8(4.45.8)	0.79	32.2(22.4-75.38)	12.0(2.6-18.8)	0.01
*IL-8(pg/ml)*									
induced	505.4(161.3-805.9)	517.0(193.3-1257.2)	0.54	233.4(170.0-1257.2)	529.0(224.5-1156.0)	0.37	277.6(170.0-649.0)	655.49(274.70-832.70)	0.32
spontaneous	221.0(165.4-674.4)	405.0(173.2-891.8)	0.42	210.1(112.7-844.1)	546.3(174.7-891.8)	0.18	336.9(210.1-734.2)	570.4(265.8-1141.4)	0.23
*IP-10(pg/ml)*									
induced	216.5(130.9-826.2)	624.3(289.9-1844.9)	0.08	232.5(175.2-748.3)	600.8(216.7-1844.9)	0.20	477.5(175.2-2554.8)	696.1(56.0-1574.1)	0.85
spontaneous	345.6(124.9-716.5)	461.0(237.5-1361.7)	0.14	354.5(129.7-780.1)	448(237.5-1202.0)	0.33	491.9(285.2-2269.7)	582.7(180.0-1093.0)	0.42
*TNF-*α*(pg/ml)*									
induced	0.4(0-1.9)	1.5(0-4.8)	0.34	0.00(0.00-2.5)	1.6(0-4.2)	0.14	2.3(0.0-5.1)	3.2(0.7-24.3)	0.24
spontaneous	0.9(0-3.2)	3.3(0.3-7.4)	0.20	0.5(0.0-4.5)	2.4(0.8-7.4)	0.18	6.43.7-14.0	7.9(2.1-173.9)	0.69
*IL-18(pg/ml)*									
induced	9.0(0.9-19.3)	8.4(4.1-25.6)	0.58	10.6(2.4-14.9)	6.3(4.1-25.6)	0.91	5.04(0.0-13.6)	4.2(0.6-10.1)	1.0
spontaneous	2.9(0.5-14.8)	14.8(8.3-31.7)	0.01	8.8(1.0-15.1)	14.6(3.5-29.8)	0.24	10.4(5.0-13.9)	7.0(2.7-25.2)	0.45
*MIG(pg/ml)*									
induced	332.2(30.1-661.7)	806.3(110.5-1391.4)	0.15	121.8(41.2-897.0)	661.7(110.5-1314.3)	0.27	157.8(43.3-1714.8)	677.1(179.0-961.8)	0.54
spontaneous	208.5(31.7-385.8)	626.8(222.6-2031.7)	0.03	280.4(69.8-1537.1)	383.4(205.3-1941.6)	0.47	1473.0 (438.8-4392.9)	740.5(384.6-2088.3)	0.33

^*^Kruskal-Wallis test.

To assess the safety of induction during exacerbations and the stable state we calculated the decline in FEV_1_% predicted during induction for all COPD patients who underwent inductions both in the BCCS and BCES. For decline in FEV_1_% predicted from post bronchodilation values during induction the relative fall was calculated (thus a fall from 30% predicted to 20% predicted will be presented as a 33% decline). To avoid repeated measurements from the same patient at steady state and/or at exacerbations only one registered induction at the two different disease states was selected for analyses per patient. 63 patients were induced during exacerbation. 33 of the patients were GOLD stage III or IV, while the remaining 30 were GOLD stage II. We found no significant difference in FEV_1_% predicted decline caused by induction related to disease severity (p = 0.07) during exacerbations. When comparing patient groups in the stable state we found that patients with more severe COPD had a statistically larger decline related to induction, than patients with COPD GOLD stage II (p < 0.001). The relative fall was significantly higher during the stable state than during exacerbations (p = 0.03) (Table 5). However, no adverse events followed inductions regardless of disease state and severity, and all patients increased in FEV_1_ after a rest period and a new inhalation of salbutamol.

**Table 5:** **Relative FEV**_**1**_ **decline in % predicted during sputum induction**

	**Exacerbations**	**Steady state**	**p** ^*^
	**n = 63**	**n = 390**	
			0.004
Median (IQR)	12.64(5.56-21.79)	18.75(11.11-25)	
Mean (SD)	14.80(13.05)	18.51(11.44)	

^*^Wilcoxon sign rank test.

## Discussion

This study showed that for the six inflammatory markers, the correlation between levels measured in induced and spontaneous sputum pairs was fair, but the agreement was quite low. TNF-α was significantly higher in spontaneous sputum samples than in induced samples when measured during a COPD exacerbation. Further, there was a relationship between HI carrier state and IL-6, and smoking status and IL-18 and MIG, found only in spontaneous sputum samples.

There are some methodological issues to consider. Firstly, it has been shown that both PBS and DTT affect the recovery of some cytokines [12,23]. However, a strength of this study was that the exact same processing protocol was used for all sputum pairs, and this should thus not impact the measured levels differently between spontaneous and induced sputum samples. Secondly, all the inflammatory markers were measured in simplex, thus the potential measurement error is greater than if the markers were measured in duplex. The choice of analysing in simplex was due to cost, since this is part of a larger analysis of inflammatory markers in sputum. Most importantly however, all sputum pairs were analysed on the same plate, on the same day. Thus the measurement error should not differ between spontaneous and induced samples. Thirdly, we found associations between inflammatory markers and smoking, and inflammatory markers and colonization with HI only in spontaneous sputum. We found no association between inflammatory markers and GOLD stage in either type of sputum, but this may be due to lack of strength. Finally, the choice on whether to induce or not during an exacerbation was based on several subjective factors in addition to oxygen saturation; most importantly patients’ willingness to be induced and the clinicians’ evaluation regarding obstructivity. Thus, it is impossible from this design to conclude that sputum induction would be safe during all exacerbations.

Although more studies on the subject of whether spontaneous and induced sputum samples could be compared was recommended already in 2002 [4], few studies have yet been published. We have found one earlier report on levels of IL-8 in spontaneous versus induced sputum that showed no significant differences in IL-8 levels between the two sputum types in COPD patients in stable state [16]. Our study confirmed the results from this earlier study, but in addition we were able to show that this is true also during exacerbations. We have been unable to find earlier reports on the relationship between levels of inflammatory markers in spontaneous and induced sputum for the remaining five inflammatory markers. To our knowledge comparison of other inflammatory markers in induced and spontaneous sputum sampled on the same consultation has not been performed in patients with obstructive pulmonary disease.

It has previously been shown that the sputum sampled early during induction has a different consistency and cell composition than sputum sampled late in the induction [24,25]. It is likely that more central airways are sampled early, and would thus most resemble spontaneous sputum. Thus, induced sputum is likely to sample a more distal airways environment than spontaneous sputum. Central and distal airways differ by epithelial components [26], distribution of immune cells [27,28], and possibly respiratory microbiome [29]. Thus, it is theoretically rather likely that levels of inflammatory markers differ between spontaneous and induced sputum samples. However, one can argue that spontaneous sputum could be a favorable alternative to induced sputum when patients find induction uncomfortable, or the safety of the induction is uncertain, and enable sampling in primary healthcare settings where induction is rarely if ever performed to our knowledge. Cell viability in spontaneous sputum has in some studies been shown to be poorer than in induced sputum samples [15,16]. Such was not the case in our samples, where viability was as good in the spontaneous samples as in the induced samples. In our study the time from collection to processing was usually very short, which could explain the high viability.

Although agreement for individual measurements was low, measuring levels of inflammatory markers in spontaneous sputum could have value for instance in serial measurements of spontaneous sputum, something our study is not equipped to assess. Also, although comparisons of inflammatory markers between spontaneous and induced sputum is invalid for some markers, they may be valid for others.

There are still sparse data on the safety on induction in patients with severe COPD during exacerbations, and in several studies sputum induction is performed during exacerbation without the published reporting on potential adverse effects on the procedure [3,30,31].

In our study we found statistical differences in FEV_1_% predicted decline between patients with moderate and severe/very severe COPD only during steady state, while disease severity did not affect the decline during exacerbations. No adverse events were registered during either the steady state or during exacerbations. This is in accordance with other reports [7,11], but we expand by including patients with severe/very severe COPD. However, it should be stressed that necessary precautions need to be taken such as having access to acute rescue medications, and that all inductions only should be performed by trained medical personnel [32].

The results from the current study point toward a necessity for reporting on sampling methods when considering inflammatory markers in sputum samples collected from COPD patients both during the steady state and during acute exacerbations as the agreement was generally low as assessed by Bland & Altman’s 95% limits of agreement. Whether levels of inflammatory markers can be compared between spontaneous and induced sputum samples likely differ by each inflammatory marker in question, and should be addressed within each study. In cases where induced sputum sampling is impossible, spontaneous samples may have value if compared with other spontaneous samples.

## Abbreviations

BCCS: Bergen COPD cohort study
BCES: Bergen COPD exacerbation study
COPD: Chronic obstructive pulmonary disease
DDT: Dithiothreitol
GOLD: Global initiative for chronic obstructive lung disease
HI: Haemophilus influenza
IL-6: Interleukin 6
IL-8: Interleukin 8
IL-18: Interleukin 18
IP-10: Interferon gamma-inducible protein-10; MIG, Monokine induced by gamma interferon
ISS: Induced sputum sampling
PBS: Phosphate-buffered saline
SSS: Spontaneous sputum sampling
TNF-α: Tumornecrosis factor-alpha
